# Genetic Diversity, Association, and Path Coefficient Analyses of Sorghum [*Sorghum bicolor* (L.) Monech] Genotypes

**DOI:** 10.1155/sci5/1611869

**Published:** 2024-12-26

**Authors:** Wedajo Gebre, Firew Mekbib, Alemu Tirfessa, Agdew Bekele

**Affiliations:** ^1^Department of Plant Science, Jinka University, Jinka, Ethiopia; ^2^School of Plant Sciences, Haramaya University, Harar, Ethiopia; ^3^Department of Sorghum Breeding, College of Agriculture, Kansas State University, Manhattan, Kansas, USA; ^4^Department of Plant Breeding, RAISE-FS, Stichting Wageningen Research (SWR) Ethiopia, Hawassa Liaison Office, Hawassa, Ethiopia

**Keywords:** diversity, genotypic association, indirect effect, phenotypic association

## Abstract

For sustainable genetic improvement of crops like sorghum, assessing genetic variability and knowing the nature and extent of the association between grain yield and yield-related traits is a prerequisite. However, there needs to be sufficient information about the genetic variability study as well as yield-related trait correlation and path coefficient analysis for sorghum accessions, especially those from southern Ethiopia. Hence, this field experiment assessed genetic variability, determined the nature and extent of phenotypic–genetic correlation, and analyzed the path coefficients among 17 quantitative traits. A total of 225 sorghum genotypes were tested using a simple lattice design with two replications at the Jinka Agricultural Research Center during the 2021 cropping season. Based on the analysis of variance, most traits showed highly significant (P 0.001) differences, suggesting genetic diversity between the genotypes. High estimates of GCV and PCV were noted for leaf width (3924.50% and 3924.50%), while the lowest GCV and PCV estimates were obtained for days to flowering and days to maturity. High heritability coupled with high GAM estimates was recorded for plant height, leaf number, leaf length, leaf width, and leaf area. The magnitudes of genotypic correlations were higher than those of phenotypic correlations for most of the studied traits, implying that the traits under study were genetically controlled. Grain yield was positively and significantly correlated with most of the traits at phenotypic and genotypic levels, indicating the presence of a strong inherent association between grain yield and other traits. Phenotypic path coefficient analysis showed that grain filling period and biomass yield exerted a high positive direct effect on grain yield. Genotypic path coefficient analysis revealed that biomass yield, grain filling period, leaf width, and days to flowering had a relatively high positive direct effect on grain yield. However, days to maturity, plant height, leaf number, leaf area, yield per panicle, straw weight, and harvest index exerted a negative direct effect on grain yield. Almost for all the studied traits, genotypic direct and indirect effects were higher than the phenotypic direct and indirect effects, indicating that the studied traits had a genetically inherited relationship with grain yield. Grain yield in sorghum can be improved through indirect selection for traits such as plant height, leaf number, leaf length, leaf width, and biomass yield. In general, the observed variability and the information obtained from this study can be used for the genetic enhancement of sorghum thereby developing high-yielding varieties.

## 1. Introduction

Sorghum is part of the grass family Poaceae, genus Sorghum Moench [1]. In northeastern Africa, specifically Ethiopia, it originated between 5000 and 7000 years ago [2], and subsequently spread throughout Africa, India, Southeast Asia, Australia, and the United States [3]. Worldwide, sorghum is one of the most important drought-tolerant C4 tropical crops. The importance of this crop lies in its resistance to drought, high-temperature stress, and low soil fertility, as well as its use as a biofuel and fiber. Sorghum plays a significant role in the human diet as a source of micronutrients (next to pearl millet) protein and energy requirements for millions mainly living in sub-Saharan Africa and Asia [4]. It is a valuable source of carbohydrates such as amylose (19.78%), starch (69.15%), and amylopectin (80.22%) [5]; protein (13.8%), ash (1.8%), oil (3.3%), and fiber (17.3%) [6]; minerals: calcium (336 mg/kg), zinc (12.0–23.0 mg/kg), iron (17.5 mg/kg), potassium (1548.5 mg/kg), and sodium (36.6 mg/kg) [7].

The high biomass production potential of sorghum reduces and compensates for carbon emissions by efficiently capturing and storing atmospheric carbon dioxide (CO2). In order to reduce future climate change impacts and provide food sustainably to a growing global population, there is a need to continue developing sorghum cultivars. It is possible to accomplish this by selecting genetically superior and resilient genotypes based upon source material diversity [8]. Properly assessing genetic diversity in sorghum genotypes would facilitate the development of new varieties with desirable characteristics. In developing the breeding strategy, genetic diversity and amenities could be maintained with knowledge of genetic differences between genetic materials. Genetic variability in agro-morphological traits is essential for the success of a plant breeding program, as it allows breeders to select superior individuals with desirable traits from a genetically diverse population [9, 10]. Plant breeders must have a good understanding of the level of genetic variability for the selection of parental genotypes with a broad genetic base for further genetic improvement. Considerable genetic variation exists in Ethiopian sorghum landraces, providing significant opportunities for improvement through breeding [11]. Studies on the diversity of Ethiopian sorghum landraces based on morphological attributes [12] and molecular markers [13, 14] showed the richness of the Ethiopian sorghum gene pool for important agronomic traits such as high lysine [15], high protein content [16], drought tolerance (postflowering), stay-green [17], grain mold resistance [18, 19], sugar cane aphid resistance [20], and resistance to Striga [21].

Genetic improvement in sorghum is highly dependent on genetic variability, heritability, and genetic advancement in the population, as well as the nature of the correlation between yield and its components [22]. Heritability is a measure of the phenotypic variance that can be attributed to genetic factors. Understanding heritability is essential for comprehending the genetic basis of traits and for making informed decisions about breeding programs to enhance the genetic potential of populations [23]. The genotypic and phenotypic coefficients of variation (GCV and PCV) offer valuable insights into the amount of variation in different characteristics. These, along with high heritability and genetic advances (GAs), are crucial for obtaining elite germplasm accessions to use in future breeding programs [24]. It is essential to thoroughly assess the genetic variability, heritability values, and genetic advancement of available germplasm for yield components. This information is of great importance for breeders to select the best genotypes for improvement [25]. Genetic diversity studies have been reported by different workers to document the extent of genetic variability among accessions [26–29] on sorghum genotypes.

Grain yield is a complex trait governed by multiple genes, requiring a comprehensive understanding of gene action [30]. Therefore, genetic control of grain yield can be indirectly achieved by studying agronomic, morphological, and physiological traits [31, 32]. Selection for a specific trait can impact others, regardless of their involvement in breeding programs. Studies on correlations between characters enable indirect selection for a quantitative trait, usually hard to select visually. It also allows us to understand how a trait can interfere with another [33]. Phenotypic and genotypic correlations are the key genetic parameters in the selection of superior genotypes and evaluating breeding strategies to be utilized [34]. The phenotypic correlation coefficient provides a measure of phenotypic association between traits while the genotypic correlation coefficient provides a measure of genetic association between traits that enable us to identify important traits to be considered for improvement programs [10, 28]. Grain yield in sorghum, like in other crops, is a complex trait determined by various components and influenced by genetic factors interacting with the environment. Understanding the correlations between different traits is particularly valuable for sorghum grain yield, as it helps breeders make informed decisions about which genotypes to pursue or discard. The correlation analysis showed the relationship between two or more series of characters [35–38]. Therefore, there is a need to conduct a path coefficient analysis, which divides the correlated variables into direct and indirect effects and visualizes the causal relationship in a more meaningful way. In addition to assessing the direct impact of one trait on another, path analysis also allows for the breakdown of correlation coefficients into direct and indirect influence components, as discussed by Boulelouah et al. [39] and Naik et al. [40]. Several reports have indicated the usefulness of path coefficient analysis in sorghum breeding programs [30, 41, 42]. This variability in correlation coefficients and phenotypic and genotypic parameters for yield and its components among different test populations and environments is a common phenomenon in agricultural research. It highlights the complex interactions between genotypes, the environment, and management practices that influence crop performance [43, 44].

In southern Ethiopia, information on genetic diversity, correlation, and path coefficient analysis of grain yield and yield-related traits in sorghum genotypes especially in lowland areas is limited. Genetic variation among most sorghum genotypes studied in the region remains to be explored based on quantitative traits for breeding. Therefore, the objective of this study was to assess genetic variability, to determine the nature and extent of phenotypic and genotypic correlations among the studied quantitative traits, and to identify the most important traits for indirect selection in future sorghum breeding programs.

## 2. Materials and Methods

### 2.1. Description of the Study Area

**Figure 1:**
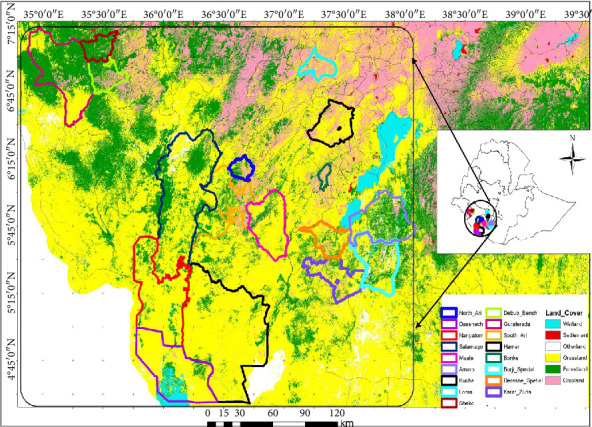
Map of Ethiopia showing the approximate areas of collection of the sorghum accession used in this study.

The field experiment was conducted from May to September 2021 at the Jinka Agricultural Research Center (JARC) during the main cropping season. The JARC is located 729 km southwest of Addis Ababa, at 360 33′ 02.7″ E, 050 46′ 52.0″ N, and an altitude of 1420 m. The maximum, minimum, and average temperatures of the center for 10 years (2012-2022) are 27.880C, 17.610C, and 22.740C, respectively, while the mean annual rainfall is 1381 mm. The soil type of the center is Cambisols [45]. The accessions studied represented all lowland areas of sorghum-growing districts in southern Ethiopia (Figure 1).

### 2.2. Experimental Materials

The experiment was executed based on 225 sorghum landrace accessions. Two hundred ten low-land sorghum accessions taken from the Ethiopian Biodiversity Institute (EBI) and 15 nationally released sorghum varieties obtained from the Melkassa Agricultural Research Center were considered (Table 1). The accessions were received from the national Ethiopian sorghum collection with full passport data and homogenized at Jinka University.

### 2.3. Experimental Design

The experimental design was a 15 × 15 simple lattice with two replications. Each genotype was planted in a plot size of 3.75 m^2^ (each row was 5 m long, 0.75 cm m between rows, and 0.15 cm between plants, and spacing between blocks was 2 m and 0.5 m between plots). Each plot had two rows, which accommodated 33 plants/seeds per row.

The seeds were sown in hand drilling, and seedlings were thinned with care after 7 days of emergence to maintain 15 cm spacing between plants. Plots were fertilized with 19 kg per hectare of nitrogen (N), 38 kg per hectare of phosphorus (P2O5), and 7 kg per hectare of sulfur (S) NPS in the form of NPS fertilizer at planting, and an additional 23 kg per hectare of nitrogen was applied in the form of urea 45 days after planting. Land preparation, weed management, pest control, and related agronomic practices were applied as per the requirement.

### 2.4. Data Collection

Sixteen quantitative traits were measured using the IBGR/ICRISAT sorghum descriptor [46]. Data were collected on days to flower (DF), days to maturity (DM), plant height (PH), grain fill duration (GFD), and leaf characteristics including leaf length (LL), leaf number (LN), leaf width (LW), and leaf area (LA). Grain yield and yield components data were collected at maturity, including panicle length (PL), panicle weight (PW), panicle yield (PY), and thousand kernel weight (TKW). The data collected on a plot basis include DF (days), DM (days from planting up to the time when 95% of plants mature (when seed texture becomes hard), straw weight (kg), biomass yield (kg), grain yield (kg), harvest index (%), and 1000 seed weight (g). The data collected on a plant basis from 10 plants were PH (cm), PL (cm), LL (cm), LW (cm), LN, threshing percent (%), and PY (g). LA (cm^2^) per plant was calculated based on the length and width of the third top leaf multiplied by the total number of leaves and a coefficient of 0.71 [47].

### 2.5. Data Analysis

The analysis of variance (ANOVA) was executed using SAS software ver. 9.1 [48], as per the following linear model for a simple lattice design. Duncan's multiple range rest (DMRT) was used for mean separation at a 5% probability level. The linear model for the simple design is as a lattice outlined and described as follows (Table 2):

(1) $$\begin{array}{c} Yijl=\mu +\text{Ti}+\text{rj}+pl\left(j\right)+\sum ijl, \end{array}$$

where *μ* = overall mean, Rj = replication effect (fixed) of the *j* th genotype, plj = random effect of block *j* within replication *i*, Tk = effect of treatment *k* (random or fixed), and ∑*ijl* = total error.

#### 2.5.1. Estimation of Phenotypic and Genotypic Variance of Components

The phenotypic and genotypic variance of components and the coefficient of phenotypic and genotypic variability were estimated based on the method suggested by Burton and De Vane [49] as follows:

(2) $$\begin{array}{c} g\text{enotypic variance}\left(\sigma_{g}^{2}\right)=\frac{{\text{MS}}_{g}-{MS}_{e}}{r},\\ e\text{nvironmental variance}\left(\sigma_{e}^{2}\right)=e\text{rror mean square}={\text{MS}}_{e},\\ p\text{henotypic variance}\left(\sigma_{p}^{2}\right)=\sigma_{g}^{2}+\sigma_{e}^{2}, \end{array}$$

where MS_*g*_ = mean square due to genotypes, MS_*e*_ = environmental variance (error mean square), and *r* = number of replications.

The PCV and GCV were estimated following the procedure of Burton and De Vane [49] and Johnson, Robinson, and Comstock [50] as follows:

(3) $$\begin{array}{c} p\text{henotypic coefficient of variation}\left(\text{PCV}\right)=\frac{\sqrt{\sigma^{2}p}}{\bar{x}}\times 100,\\ g\text{enotypic }c\text{oefficient of variation}\left(\text{GCV}\right)=\frac{\sqrt{\sigma^{2}g}}{\bar{x}}\times 100, \end{array}$$

where $\bar{x}$ = grand mean of traits, *σ*_*p*_^2^ = phenotypic variance, and *σ*_*g*_^2^ = genotypic variance.

#### 2.5.2. Estimation of Broad-Sense Heritability and GA

Broad-sense heritability (H) is expressed as a percentage of the ratio of the genotypic variance (*σ*_*g*_^2^) to the phenotypic variance (*σ*_*p*_^2^) and was estimated on genotype mean base as described by Allard [51] and Falconer [34] as follows:

(4) $$\begin{array}{c} \text{Heritability}\left(h^{2}b\right)=\frac{\sigma^{2}g}{\sigma^{2}p}\times 100, \end{array}$$

where *h*^2^*b* = heritability in broad sense, *σ*_*g*_^2^ = genotypic variance, and *σ*_*p*_^2^ = phenotypic variance = *σ*_*g*_^2^ + *σ*_*e*_^2^.

GAs in the absolute unit and percent of the mean (GAM), assuming the selection of a superior 5% of the genotypes, were estimated following the methods illustrated by Johnson, Robinson, and Comstock [50].

(5) $$\begin{array}{c} GA=K*\sigma_{p}*H, \end{array}$$

where *K* = the standardized selection differential at 5% selection intensity (*k* = 2.063), *σ*_*p*_*p* = phenotypic standard deviation on mean basis, *H* = heritability in broad sense.

GA as a GAM was calculated to compare the extent of the predicted advance of different traits under selection, using the formula described by Comstock and Robinson [52]:

(6) $$\begin{array}{c} \text{GAM}=\frac{GA}{\overset{¯}{X}}\times 100, \end{array}$$

where GAM = GA as percent of mean, GA = genetic advance under selection, $\overset{¯}{X}$ = mean of the population in which selection employed

#### 2.5.3. Correlation and Path Coefficient Analysis

The collected data were subjected to ANOVA to test the presence of variation among genotypes for the studied traits. Based on the ANOVA results, all significant traits were used for correlation and path coefficient analysis. Grain yield is a complex trait affected by the interactive effects of several yield-associated traits that might be predisposed to by their genetic structures and also other environmental factors. Thus, the direct measurement and improvement of grain yield may be ambiguous due to the influence of environmental factors and the nature of the genetic makeup of the trait. Therefore, it is important to investigate factors that affect grain yield and related traits. To this end, estimating genotypic and phenotypic correlations is a vital tool to understand their influence in advance. Phenotypic and genotypic correlations between yield and yield-related traits were estimated using the method outlined by Miller et al. [53]:

(7) $$\begin{array}{c} t\text{he phenotypic correlation coefficient}\left(rp_{xy}\right)=\frac{Covp_{xy}}{\sqrt{Vp_{x}Vp_{y}}},\\ g\text{enotypic correlation coefficient}\left(rg_{xy}\right)=\frac{Covg_{xy}}{\sqrt{Vg_{x}Vg_{y}}}, \end{array}$$

where *r*_*pxy*_ = phenotypic correlation coefficient, *r*_*gxy*_ = genotypic correlation coefficient between traits *x* and *y*, Cov_*pxy*_ = phenotypic covariance, and Cov_*gxy*_ = genotypic covariance between traits *x* and *y*, while *Vp*_*x*_ = phenotypic variance for traits *x*, *Vp*_*y*_ = phenotypic variance for traits *y*, *Vg*_*x*_ = genotypic variance for traits *x*, and *Vg*_*y*_ = genotypic variance for traits *y*, respectively.

The significance of the coefficient of correlation at the phenotypic level was tested by comparing the correlation coefficient with tabulated *r*-values at 0.05 and 0.01 probability levels for g-2 degrees of freedom. However, the coefficient of correlation at the genotypic level was tested for significance using the formula described by Robertson [54]:

(8) $$\begin{array}{c} t=\frac{\left(rg_{xy}\right)}{SEg_{xy}}, \end{array}$$

where the calculated' value was compared with the tabulated' value at g-2 degree of freedom at 5% level of significance.

(9) $$\begin{array}{c} SEg_{xy}=\sqrt{\frac{\left(1-r^{2}g_{xy}\right)}{2h_{x}\cdot h_{y}}}, \end{array}$$

where *SEg*_*xy*_ = standard error of genotypic correlation coefficient between traits *x* and *y*, *h*_*x*_ = heritability value of traits *x*, *H*_*y*_ = heritability value of traits *y*, *r* = correlation coefficient *x* and *y*, and *g*_*xy*_ = value of traits *x* and *y*, respectively. The absolute *t*-value we calculated was compared to the tabulated *t*-value at degrees of freedom (*g* − 2) for both phenotypic and genotypic correlations. The environmental correlation coefficients were tested at degrees of freedom [(*g* − 1)(*r* − 1) − 1], where *g* represents the number of genotypes as defined by Robertson [54]. META-R Version 6.01 [55] was employed for phenotypic and genotypic correlation coefficient analyses.

Microsoft Excel computer program was employed for phenotypic and genotypic path coefficient analyses as well as estimation of residual effect.

Path coefficient analysis was conducted as suggested by Dewey and Lu [56] using the phenotypic as well as genotypic correlation coefficients to determine the direct and indirect effects of yield components on grain yield based on the following relationship:

(10) $$\begin{array}{c} R_{ij}=P_{ij}+{å}_{rik}P_{kj}, \end{array}$$

where *R*_*ij*_ = mutual association between the independent traits (*i*) and dependent traits, grain yield (*j*) as measured by the correlation coefficients; *P*_*ij*_ = components of direct effects of the independent traits (*i*) as measured by the path coefficients; and å_*rik*_*P*_*kj*_ = summation of components of indirect effect of a given independent traits (*i*) on a given dependent traits (*j*) via all other independent traits (*k*).

The contribution of the remaining unknown factor was measured as the residual factor (PR), which is calculated as follows:

(11) $$\begin{array}{c} \text{PR}=\sqrt{\left(1-\sum r_{ij}p_{ij}\right)}. \end{array}$$

The magnitude of PR indicates how best the causal factors account for the variability of the dependent factor [57].

## 3. Result and Discussion

### 3.1. Analysis of Variance

The ANOVA results showed significant differences among sorghum genotypes for all the studied traits except PL (Table 3). A similar result was reported by Karp et al. [58] on twenty-seven rabi-colored sorghum genotypes. The presence of a considerable range of variability among the genotypes for the traits under study is necessary to identify superior genotypes and would also provide a good opportunity for the improvement of this crop through the selection of useful traits. The observed genotypic and phenotypic variances among the genotypes suggest that adequate genetic variability can be beneficial for improving yields through selection. This finding aligns with previous results [35, 59, 60], which indicated that the presence of variability in certain studied traits among the sorghum genotypes suggests ample opportunities for the selection of these traits. Similarly, Mulualem et al. [41] reported similar results in 110 sorghum genotypes, Tesfaye [61] in 119 sorghum genotypes, and Ullah et al. [62] in 30 sorghum accessions. Likewise, Vanipraveena, Talekar, and Kachapur [63] observed significant differences among 52 sweet corn genotypes for DF, DM, PH, ear height, ear length, number of kernel rows per year, total soluble solids, and green ear yield. Similar results were reported by several authors [64–67] for days to 50% flowering, DM, PH, panicle width, LL, number of leaves per plant, hundred-seed weight, yield per plant, and PW. Similar results were recorded by Chavhan, Jawale, and More [68] on B parental lines of Kharif sorghum genotypes and Thamizhiniyan et al. [69] on red sorghum genotypes.

### 3.2. Simple Measure of Variability

A comprehensive estimate of the mean, range, genotypic and phenotypic variances, heritability in a broad sense, GA, GAM, and standard errors of 225 sorghum genotypes is presented in Table 3. The genotypes showed high variability in PH, LN, LL, and LA. Therefore, the present finding showed the presence of inherent genetic variability among the sorghum genotypes, suggesting a good opportunity for the selection of genotypes with desirable traits for further improvement. The existence of variability is important for improving those traits through direct phenotypic selection, thus suggesting the presence of additive gene effects. Similar findings were presented by Mofokeng et al. [70] for 98 sorghum accessions, Kavipriya et al. [71] for 80 sorghum landraces, and Adedugba et al. [72] for 112 sorghum accessions.

There was a significant variance in DF among the genotypes (Table 3). The mean values ranged from 63 to 100 days with an overall mean of 77.24 (Table 4). The majority of genotypes exhibited medium flowering, with a few genotypes showing early flowering. The genotypic variance was significant due to the DM, and the means ranged from 110 to 152.5 days with an overall mean of 133.83 days (Table 4). Grain filling period variability ranged from 23.5 to 82 days with a mean of 56.58 days. Senbetay [73] also reported a wide range of variability among sorghum genotypes about DF (65–112 days) and DM (141–164 days). The present study showed that the majority of the genotypes attained their maturity earlier than most of the standard checks, suggesting a potential for obtaining drought-escaping materials from the tested genotypes in drought-prone areas of the country through simple phenotypic selection. Gichile [10] proposed that early maturity could be advantageous for selection in drought-stressed environments with short and irregular rainy seasons. In Ethiopia, climate change has led to progressively shorter rainy seasons [74] and has caused a transition from cereal to other crops to adapt to the adverse effects [75]. Hence, the selection of early flowering and maturing genotypes is highly recommended despite the potential yield penalty [76].

The ANOVA results depicted that there were significant variations observed among the genotypes for PH which ranged from 75 to 393 cm, with a mean of 225.79 cm. PH is an important character used in the selection of genotypes for different purposes. For instance, tall genotypes are preferred for fodder production, fuel, and house construction [77] while short PH has been identified as an important trait for drought tolerance [78, 79]. Yield arbitrating traits such as PL, yield per panicle, PW, TKW, and harvest index showed wide variability that could be exploited in cultivar development through selection. This finding is consistent with research by Naoura et al. [66], who noted a significant amount of variability in PH, PW, and TKW. As shown in (Table 4), the ANOVA results depicted that there were significant differences observed among the genotypes for straw weight, biomass yield, and grain yield. The values ranged from 768.1 to 5725 (kg ha^−1^), 3338–14012 (kg ha^−1^), and 1613–11511 (kg ha^−1^) with a mean of 2245.51 (kg ha^−1^), 8250.26 (kg ha^−1^), and 6004.62 (kg ha^−1^), respectively. Concerning leaf characteristics, the tested genotypes also showed a wide range of variability for LN (6–14), LL (38–98 cm), LW (0.5–12 cm), and LA (14.2–673.8 cm^2^). The present study showed the presence of a wide range of genetic variability among studied sorghum genotypes for grain yield and yield-related traits.

### 3.3. Estimations of Genetic Parameters

#### 3.3.1. Estimates of Variance Components

The characteristics of genetic variation, such as genotypic variance, phenotypic variance, GCV, PCV (%), heritability, and GA, are presented in Table 4. The GCV and PCV values for DF, DM, grain filling period, PH, LL, LN, LW, LA, PL, yield per panicle, PW, straw weight, biomass yield, TKW, grain yield, harvest index, and threshing percent are listed in Table 4. Similar results have been reported by Ullah et al. [62] for DF, DM, PH, TKW, and grain yield; Swamy et al. [78] for DF, DM, PH, PL, PW and yield, and TKW; Subramanian, Raj, and Elangovan [80] for DF, PH, leaves, LL, PL, and PY. The study by Patil et al. [81] focused on DF, DM, PH, and yield per panicle. Also, Meron, Alemu, and Taye [82] reported similar results on PH, LN, LA, and biomass yield. These findings suggest potential for further improvement of these traits through simple selection. Based on the analysis of GCV and PCV in sorghum genotypes, it was found that there is significant variability in almost all of the studied traits (Table 4). This indicates the presence of broad genetic variation among the genotypes, a conclusion that is consistent with previous research on sorghum [83–85].

PCV and GCV values were assumed to be low (0%–10%), moderate (10%–20%), and high (greater than 20%) [86]. The result of variance components, GCV, and PCV of the traits exhibited that the magnitude of GCV and PCV was maximum for LW, LA, PH, and straw yield, indicating that these traits had wide genetic variability and would respond better to selection. Moderate estimates of GCV and PCV were observed for traits such as grain filling period, LN, LL, PW, biomass yield, grain yield, and threshing percent. Low GCV and PCV were recorded for DF and DM. A low GCV indicates that the improvement of this trait through selection would be less effective due to lack of genetic variability, while a low PCV indicates that these traits contribute little heritable genetic (additive) factors to future generations, so there is no need to invest in improving them to improve sorghum. The lower GCV and PCV values in the current study are in agreement with the findings reported by Ranjith et al. [87] and Tilaye Tadesse [88]. Likewise, Fikre, and Alamerew [89]; Alemu, Firew, and Tadesse [90]; and Chewaka [91] reported low estimates of GCV and PCV for days to 50% flowering and DM. Therefore, there is little scope for further improvement of these traits through simple selection, but it preferably indicates that there is considerable possibility of further improvement through crossing followed by appropriate selection for these characters.

In this study, the PCV values for all traits were higher than the GCV values, indicating that environmental factors have a greater influence on these traits. This finding is in agreement with previous reports on sorghum genotypes [25, 38, 84, 92–94]. Likewise, Elangovan and Bahadure [95] reported that the magnitude of PCV was higher than GCV for dry fodder yield per plant followed by PW per plant, grain yield per plant, and PL, respectively, on 203 sorghum genotypes.

Estimates of GCV ranged from 1.75% for PL to 3924.50% for LW. PCV values ranged from 8.74% for DM to 3924.50% for LW. This finding aligns with the report by Elangovan and Bahadure [95] and Nirosh et al. [96] who observed wide differences between PCV and GCV in traits such as DF, DM, PH, PL, PW, yield per panicle, and TKW indicate their susceptibility to environmental fluctuations. For all traits studied, high genetic variability was observed among sorghum genotypes. The observed variability was the sum of variation arising due to genotypic and environmental effects. As a result, knowledge of the nature and magnitude of genetic variations contributing to the genetic gain under selection is essential.

Among the traits that showed the maximum differences between GCV and PCV, the largest values were found for LW and LA, indicating that the expression of these traits was more influenced by the environment. Nevertheless, the minimum differences were observed for DF followed by DM, suggesting that these traits are less influenced by environmental factors and more influenced by genotypic factors or fixable genes. This signifies that the variability in these traits is mainly due to genetic differences, with less influence from the environment. Therefore, selection based on the phenotype alone can be effective for the improvement of these traits.

### 3.4. Estimates of Heritability and GA

The estimates of broad-sense heritability (*h*^2^*b*) and GA for various traits of sorghum genotypes are shown in Table 4. The lowest estimates of heritability were observed for panicles while the highest was observed for LW (Table 4). Heritability values act as predictive tools in expressing the real potential of phenotypic value; hence heritability values are used to predict the expected progress to be achieved through the process of selection. As reported by Hanson, Robinson, and Comstock [97], heritability is generally classified as low (0%–30%), moderate (30%–60%), and high (60% and above). Higher magnitudes of heritability were observed for LW, LA, LN, LL, and PH. Mohammed, Bulti, and Girma [98] correspondingly reported higher magnitudes of heritability were observed for LW, LA, LN, LL, and PH on sorghum genotypes which agrees with the earlier reports of [82, 94, 99] on sorghum.

In the current study, heritability estimates of greater than 30% were recorded for LW, LA, LN, LL, PH, and straw weight. This suggests that traits with high heritability estimates are more likely to be passed down from one generation to the next, with genetic factors playing a larger role in determining the variability of those traits. In contrast, traits with low heritability estimates are more likely to be influenced by environmental factors, such as upbringing, diet, and lifestyle. Therefore, these highly heritable traits are expected to remain stable under different environments, as the environment is less influential and could easily be improved through selection pressure. A similar result obtained by Jain and Patel [100], indicated that there were high heritability values observed for the studied traits, indicating that the variation arises due to additive genetic effect, which coincides with the previous works of several researchers [66, 101–104] on sorghum, who observed high heritability percentages among the tested genotypes. In general, traits having high heritability estimates were mainly controlled by additive types of genes while those traits with low heritability indicate those characters are highly influenced by environmental factors and influenced by nonadditive types of genes.

Although estimates of high heritability alone will not ensure the amount of gain through selection, rather heritability estimates with GAM considered together can help to conclude the nature of gene action governing particular traits. Jain and Patel [100] indicated that high heritability with high GA may be presumed that the genotypic variance for these traits was mainly due to additive genetic effect and selection based on phenotypic performance could be effective in achieving desired results. Estimates of GA, as a percent of mean ranged at 5% selection intensity, ranged from 0.06% for harvest index to 1072.36% for biomass yield per hectare (Table 4). According to Johnson, Robinson, and Comstock [50] and Falconer and Mackay [23], GMA percent was considered low (< 10%), moderate (10%–20%), and high (> 20%). A genotype's heritability alone does not provide a true indication of its genetic potential due to the interaction between genotype and environment.

High GA coupled with high heritability was observed for PH, LN, LL, LW, and LA (Table 4). Zarea, Deshmoukh, and Smail [105] similarly observed high heritability estimates coupled with high GA as a percent of the mean for the trait PH on 14 sorghum genotypes, indicating the impact of additive gene expression which coincides with the earlier reports by the authors of [101, 103, 106–108], for PH, LN, LL, LA, LW, LA, and straw weight on sorghum. Similarly, Prasad and Sridhar [109] observed high heritability and high GA for PH and straw weight. High heritability coupled with high GA as a percent of the mean was reported by Singh, Gangwar, and Chaudhary [110] for LA and green fodder yield per plant.

Grain filling period, PY, straw weight, biomass yield, grain yield, and threshing percent showed moderate heritability and moderate GAM. The findings are consistent with those of Adedugba et al. [72] who observed that moderate heritability coupled with moderate GA as a percent mean for straw weight on one hundred and twelve accessions of sorghum, indicating that the expression of traits is more likely to be influenced by environmental factors and controlled by nonadditive gene action. Similar results were reported in sorghum [102, 111, 112].

### 3.5. Genotypic and Phenotypic Associations Coefficient Analysis

The correlation coefficients among grain yield and its contributing traits were computed, and the values for phenotypic and genotypic correlation coefficients between each pair of traits are presented in Table 5. As suggested by Oliveira et al. [113], the coefficients of Pearson's correlation were classified as weak (*r* from 0.10 to 0.30), moderate (*r* from 0.40 to 0.60), or strong (*r* from 0.70 to 1). The current study revealed that the magnitudes of genotypic correlation coefficients (*rg*) were higher than their corresponding phenotypic correlation coefficients (*rp*) except in a few cases, indicating the presence of strong inherent associations among these traits due to genetic factors and the dominance of genetic variance in the expression of traits. The presence of small values for the genotypic correlation coefficient could hinder the speed of selection progress. Negative values of genotypic coefficient correlation were found by some of the traits indicating that the selection for some of the traits would affect each other in the opposite direction as earlier findings of Maiga et al. [114]. This finding coincides with the previous works of Premkumar, Nirmalakumari, and Anandakumar [115] on oats [28, 116], on sorghum [89, 117], on wheat, and [118, 119] on rice. These authors reported that the magnitudes of genotypic correlation were higher as compared to their corresponding phenotypic correlation for most of the traits indicating the preponderance of genetic variance in the expression of characters. On the contrary, Tabasum, Saleem, and Aziz [120], Amare, Zeleke, and Bultosa [25], Ahalawat et al. [121], and Khandelwal et al. [122] reported that the magnitude of phenotypic correlation was higher than genotypic correlation. Also, Thant et al. [123] and da Silva et al. [124] reported that the magnitude of phenotypic correlation was higher than genotypic correlation. A similar study by Dev et al. [101] indicated that the PCV for 10 characters on 10 forage sorghum genotypes was larger than the genotypic coefficient, which suggests that both genotype and environment are involved in variation which suggests that genotype × environment interaction is an important factor to consider when attempting to understand variation in forage sorghum.

Almost all yield components showed a significantly positive correlation with grain yield except PL at the genotypic and phenotypic level, suggesting that genotypes with larger and wider panicles, more primaries per panicle, grain yield per panicle, and hundred seed weights produced a higher yield. Therefore, these traits are important for increasing sorghum grain yield. These findings are in lined with Premkumar, Nirmalakumari, and Anandakumar [115], Alemu and Demelash [125], Bibi et al. [126], and Pavan Kumar and Biradar [127]. The occurrence of a genotypic correlation between two traits can happen due to pleiotropy or gene dosage imbalance. Pleiotropism is one of the causes of high correlations since one gene can influence the expression of more than one trait.

Finally, the genetic relationships of a character may be linked to the presence of pleiotropic effects of a gene, linkage of two traits, linkage disequilibrium, and epistatic effects of different genes, or it may be due to environmental influences [23, 51].

#### 3.5.1. Associations of Grain Yield With Other Traits

Grain yield exhibited significant and positive correlations with yield per panicle, PW, straw weight, biomass TKW, harvest index, and threshing percent at the genotypic and phenotypic levels (Table 5). This indicates that these traits are considered important for the improvement of sorghum yield through direct selection of these traits. This result is in agreement with the work of Bhandari et al. [119] on rice, who reported that the selection for those traits having strong and positively significant correlations with grain yield is expected to improve grain yield in sorghum, suggesting that the true relationship of these traits with grain yield since these traits are grain yield predictors. Similarly, Shivaprasad et al. [128] indicated that traits that have moderate to strong correlations with the desired trait are important for successful indirect selection.

Regarding the intercorrelations between different traits, LW had a positive and significant correlation with DF, DM, and LL. This is in agreement with the reports by Vinodhini et al. [129] on the sorghum genotypes, who indicated that the strong and positive association of yield-related traits might be due to the linkage of genes determining these traits which leads to the simultaneous improvement in grain yield through these traits could be achieved within a short period by simple selection procedures. Therefore, these results give some clues as to how simultaneous improvement in any of these traits will lead to an increase through direct selection of grain yield in sorghum. Based on these results, the genotypic and phenotypic correlations indicate the extent to which the corresponding traits are under the control of the same set of genes having a physiological basis for their expression. Likewise, Thant et al. [123] on sorghum genotypes reported that grain exhibited a significantly (*p* ≤ 0.01) positive correlation with TKW and stover yield at the genotypic (0.453, 0.504) and phenotypic (0.453, 0.480) levels, respectively, while it had exerted significantly (*p* ≤ 0.05) positive correlation with the number of leaf breath only at the genotypic (0.291) level. Similarly, grain yield per panicle showed a significant (*p* ≤ 0.05) positive correlation with PW, biomass, harvest index, and threshing percent at the genotypic and phenotypic levels. On the other hand, grain yield showed a negative and significant genotypic correlation with LW at the genotypic level (Table 5).

Genetic relationships between grain yield and yield-related characters are prerequisites for selecting desirable types for the target environment. Some of the yield components are highly interrelated while grain yield is governed by many genetic as well as environmental factors that are interdependent and influenced by various components of yield, which is associated with low heritability. Investigations into correlations between characters are indeed important to breeding programs as they enable indirect selection for a quantitative trait, usually hard to select, by another directly correlated trait of higher genetic gain or easy visual selection; as well, it is also able to access how a trait can interfere with another [129]. It is known that the selection for a certain trait can eventually affect others with or without interest in the intended breeding programs. Accordingly, understanding of correlations between traits is practical for grain yield since it allows the breeder to use that additional information to reject or promote genotypes of interest. In general, the current study suggested that the presence of a strong inherent association among the studied traits leading to direct phenotypic selection might be worthwhile for the improvement of sorghum which implies that there is an enormous chance of exploiting the potential of these traits for effective selection in a sorghum improvement program.

### 3.6. Path Coefficient Analysis

Whether the correlation between two characters is phenotypic, genotypic, or environmental and due to the influence of a third factor on the association between two variables. To understand the nature of correlations among yield and yield components to improve production and mitigate future demand, it is necessary to examine the cause-and-effect relationship between dependent and independent variables. By considering this view, path analysis is a means of separating the correlation coefficient into direct and indirect effects and offers information on the real influence of a trait on yield [115]. Regarding the values for direct and indirect effects, Kovačević et al. [130] suggested that a scale for the importance of direct and indirect effects classified as negligible, for values from 0.00–0.09, low, for values from 0.10–0.19, moderate, for values from 0.20–0.29, and high, for values from 0.30–0.99, while values greater than 0.99 were considered as very high. In this study, path coefficient analysis was carried out at phenotypic and genotypic levels (Tables 6 and 7). Grain yield was considered as a dependent character while the rest of the variables that were positively correlated with grain yield were used as causal characters of the 16 characters on grain yield.

#### 3.6.1. Phenotypic Path Coefficient Analysis of Grain Yield With Other Trait

The phenotypic path coefficient analysis results of grain yield with the other 16 traits are presented in Table 6. The path coefficient analysis results revealed that DF, grain filling period, PH, LN, LL, LA, PL, and biomass yield had a positive direct influence on economic yield at the phenotypic level. The grain filling period had a maximum direct positive effect on grain yield (1.34) followed by biomass yield (1.15), suggesting that the simultaneous selection of the two traits may improve genetic gain in sorghum breeding. PH possessed a positive direct effect (0.40) on grain yield, but its negative indirect effects were through LL (−0.01), LA (−0.00), yield per panicle (−0.02), and TKW (−0.004). The results showed that PW, TKW, harvest index, and threshing percent followed by LL and LN were found to have negligible contributions either in positive or negative directions to grain yield, indicating the selection of these characters would be less effective for improving grain yield. In this study, those traits with positive direct effects should be considered as selection criteria for yield improvement in sorghum, and revealing the effectiveness of direct selection through these traits is important. Direct negative effects on grain yield were attributed to days of maturity, LW, yield per panicle, and straw weight at the phenotypic level, indicating that improving these key traits related to grain yield is important before choosing them for high grain yield. Therefore, the results indicated that these traits are not directly associated with yield hence selection directly through these characters results in poor selection, so indirect causal factors must be considered especially the traits contributing positively.

DM had a negative direct effect (−1.0) on grain yield because of its negative indirect effect through grain filling period, PH, LN, LL, LA, PL, PW, TKW, and threshing percent. This finding is contrary to the results of Thamizhiniyan et al. [69] and Vinodhini et al. [128] who reported that DF showed a negative direct effect on grain yield at the phenotypic level. LW exerted a negative direct effect (−0.3) on grain yield due to its negative indirect effects through DF, DM, grain filling period, PH, LN, LL, and biomass yield. Yield per panicle exerted a negative direct effect (−7.60E − 05) on grain yield due to its negative indirect effects through DF, grain filling period, LW, PL, and biomass yield. Straw weight exerted a negative direct effect (−0.34) on grain yield due to its negative indirect effects through the grain filling period and biomass yield.

The grain-filling period affected grain yield positively and indirectly through LN, PL, biomass yield, and harvest index whereas the negative indirect effect of LL on grain yield was observed through DF, DM, PH, LN, LW, TKW, and harvest index at the phenotypic level. Similarly, a negative indirect effect of biomass yield on grain yield was noted through DF, LW, LA, yield per panicle, and PW at the phenotypic level. The considerable indirect effect of LA through DM was counterbalanced by the positive direct effect of LA on grain yield and reduced the correlation coefficient. PL affected grain yield positively and indirectly significantly via LL and width, and others (grain filling period, PH, LA, and biomass yield). Biomass yield had influenced positively and indirectly grain yield via PH, LA, and TKW at the phenotypic level. Likewise, the negligible positive direct effect of PW, TKW, harvest index, and threshing percent on grain yield was noted at the phenotypic level. PW had influenced positively and indirectly grain yield through LN, length width, and area; TKW had influenced positively and indirectly grain yield through DF, LN, LA, and biomass yield; harvest index had influenced positively and indirectly grain yield through DM, LN, LA, straw weight, and threshing percent at the phenotypic level. The result of phenotypic path analysis showed that due importance should be given to grain filling period, biomass yield, DF, and because of their significant correlation and high direct effects. This indicates that there is always scope for the enhancement of grain yield by the selection of these traits at the phenotypic level.

As suggested by Saini et al. [131], the residual effect in path analysis determines how best the component (independent) variables account for the variability of the dependent variable, which is grain yield. The residual effect at the phenotypic level was relatively low indicating that the traits considered in this study are enough to adequately explain the variation in grain yield. About 79.41% of the total variation in grain yield was contributed by 16 independent traits that were included in this study. The remaining 20.59% is explained by other traits not considered in the study. This further clarified that the grain yield contributing traits included in this study were good enough. It is also suggested that further study should be made with more characters to find out other traits that contribute to the rest of the proportion of yield. Generally, the present investigation suggested that maximum emphasis should be given to the phenotypic traits studied in selecting sorghum genotypes with higher grain yield. The contribution of residual effects influencing grain yield was minimal at the phenotypic levels, indicating that the study traits sufficiently explained the variability in the dependent character. This finding was supported by previous reports on sorghum [128, 132–134]. Similarly, Vinodhini et al. [135] observed the phenotypic residual effect, indicating that about 72% of the phenotypic total variation was explained by the included traits and how best the causal factors account for the variability of the dependent factor on red sorghum genotypes.

Therefore, path analysis indicated that PH, number of leaves per plant, LL, LW, PW, straw weight, biomass yield, harvest index, and trashing percent were the main determinants of grain yield. Thus, more emphasis should be given to the selection of these traits for yield improvement in sorghum. Improvement in grain yield in sorghum could be brought through the selection of component characters such as the number of leaves per plant, LL, LW, PW, straw weight, biomass yield, and harvest index which are directly related to the final yield in sorghum and have exhibited positive direct effects at the phenotypic level.

#### 3.6.2. Genotypic Path Coefficient Analysis of Grain Yield With Other Traits

The genotypic path coefficient analysis results for grain yield with the other 16 traits are presented in Table 7. DF, grain filling period, LL, LW, PW, biomass yield, and trashing percent had a positive direct effect on grain yield. Biomass yield possessed a high positive direct effect on grain yield, but its negative indirect effects were also detected through LW. The grain filling period exerted a positive direct effect on grain yield, but its negative indirect effects were through DF, TKW, harvest index, and threshing percent.

The results showed that biomass yield had a maximum direct positive effect on grain yield followed by grain filling period, LW, and DF, respectively, suggesting that the simultaneous selection of these traits may improve genetic gain in sorghum breeding. The strong indirect effect of biomass yield through PL, straw weight, yield per panicle, LA, and DM was counterbalanced by the positive direct effect of biomass on grain yield and reduced the correlation coefficient. Similarly, the indirect effect of PL through DM, PH, LN, PW, TKW, and threshing percent was counterbalanced by the direct effect of biomass yield on grain yield and reduced the correlation coefficient. The positive direct effects of biomass yield and PL on grain yield were diluted due to their negative indirect effects. Selection of these traits except biomass yield and PL would be highly effective for improving grain yield. Direct negative effects on grain yield were attributed to DM, PH, LN, LA, yield per panicle, straw weight, and harvest index, which indicated that the improvement of these traits is essential before selecting them for high grain yield. The negative direct effect of DM on grain yield was nullified by its positive indirect effects via grain filling period, LL, yield per panicle, PW, TKW, harvest index, and threshing percent.

LA had exerted a negative direct effect on grain yield due to its negative indirect through DM and grain filling period. The negative direct influence of DM on grain yield was nullified by its positive indirect effects via LW, yield per panicle, panicle width, TKW, and harvest index. The grain filling period exerted a positive direct effect on grain yield due to its positive indirect effects through PH, LL and width, PL, and biomass yield. Yield per panicle exerted a negative direct effect on grain yield due to its negative indirect effects through PH, LN, straw weight, TKW, and harvest index. Straw weight exerted a negative direct effect on grain yield due to its negative indirect effects through DM, PH, LN, and PW. The harvest index exerted a negative direct effect on grain yield because of its negative indirect effects through yield per panicle and TKW [136].

DM exerted positive and indirect effects on grain yield through LW, yield per panicle, PW, TKW, harvest index, and threshing percent. The yield per panicle exhibited positive and indirect effects on grain yield through DF, DM, LA, and PL at the genotypic level. This result is in covenant with other reports [35, 137]. Selection based on yield per panicle would increase grain yield indirectly via PL. Path coefficient analysis also revealed that path coefficient analysis also revealed that PL had a positive direct effect on grain yield. This trait also recorded a strong positive genotypic correlation with PW. This indicated that the selection of genotypes having long panicles along with optimum PW would be rewarding for identifying high-yielding genotypes in this crop. This is in agreement with Derese et al. [138] on sorghum, who reported that PL showed a low but positive direct effect on grain yield followed by green leaves at physiological maturity and DM, which coincides with similar works [118, 139, 140], that reported the positive direct effect of PL on grain yield of sorghum. Yield per panicle exerted a negative direct effect on grain yield. This result is controversial with previous reports [112, 124, 138] which described that grain weight per panicle exerted a positive direct effect on the grain yield of sorghum.

Positive indirect effects of straw weight were observed through LA, PL, TKW, and harvest index at the genotypic level. Likewise, a positive indirect effect of straw weight on grain yield was observed through LA, PL, TKW, and harvest index at the genotypic level. Positive indirect effects of biomass yield were observed through DF, grain filling period, PH, LL, LN, PW, TKW, harvest index, and threshing percent at the genotypic level. Harvest index affected grain yield positively and indirectly through DF, DM, PH, LN, LA, PL, PW, straw weight, biomass yield, and TKW. This result confirmed previous findings [89, 137, 141]. The results of genotypic path analysis showed that due importance should be given to biomass yield, grain filling period, and LW because of their significant correlation and high direct effects. This indicates that there is always scope for the enhancement of grain yield by selection of these traits at the genotypic level. Biomass yield, grain filling period, LW, and DF are the most important yield-contributing components as they recorded high direct effects on grain yield in sorghum.

The residual effect at the genotypic level was low, signifying that the traits considered in this study are enough to adequately explain the variation in grain yield (Table 7). Singh and Chaudhary [57] reported that if the residual value is small (for instance, nearly zero), then the dependent trait considered (grain yield) is fully explained by the other independent traits. The genotypic path showed more distinct direct and indirect effects than the phenotypic path. About 84.77% of the total variation in grain yield was contributed by 16 independent traits that were included in this study, while other factors not included in the study might explain 15.23%. Therefore, the traits included in this study were good enough to explain the variability in grain yield. Generally, the low residual effect indicated that the independent traits explained the dependent trait (grain yield) more than two-thirds (2/3); therefore, genotypic path coefficient analysis-based selection for sorghum improvement is very appropriate. This finding is supported by the report of Maiga et al. [114], who observed a low genotypic residual effect. Likewise, Pavan Kumar and Biradar [127] observed a low residual effect and stated the appropriateness of the traits chosen explained the variability. Similarly, Gobezayohu et al. [142] observed the genotypic residual effect, indicating that about 83% of the genotypic total variation was explained by the included traits and how best the causal factors account for the variability of the dependent factor on sorghum genotypes.

This result is contradictory to the reports of Lemma Tulu [94] and Mulualem et al. [41], who reported relatively high residual effects observed at the genotypic level which may be due to the weather conditions during the growing season indicating that some of the characters influencing the grain yield of sorghum have not been included in the present investigation.

## 4. Conclusions

A wide range of variability in physical characteristics was observed across all the traits that were studied. Maximum GCV and PCV were recorded for LW and LA, followed by PH and straw weight, whereas DF and DM had the lowest genotypic and phenotypic variances. GCV is more useful for assessing variability as it depends upon the heritable portion of variability. Therefore, there is a chance for the selection of the majority of the traits in the genotypes.

High estimates of heritability coupled with high GA as a percent of the mean were recorded for PH, LN, LL, LW, and LA. Therefore, the presence of high heritability values coupled with high GA as a percent of the mean for these traits suggests that the preponderance of additive gene action with low environmental influence and the improvement of these characters could be effective through direct phenotypic selection. Thus, there is an incredible opportunity for the improvement of sorghum genotypes. LW, LA, LN, LL, and PH showed high heritability values which are believed to be governed by additive gene actions and the selection for their improvement could be effective. High GA coupled with high heritability was observed for PH, LN, LL, LW, and LA.

LW had a positive and significant correlation with DF, DM, and LL at the genotypic level. Grain yield per panicle showed a significantly positive correlation with PW, biomass, harvest index, and threshing percent at the genotypic and phenotypic levels. On the other hand, grain yield showed a negative and significant genotypic correlation with LW. A positive correlation was noted for DF, PH, LN, LA, and TKW while DM, grain filling period, LL, LW, and PL were correlated negatively with grain yield at the phenotypic level.

All yield components showed a significantly positive correlation with grain yield except PL at genotypic and phenotypic levels. A significant and positive correlation was recorded between grain yield and grain yield per panicle was noted at phenotypic levels. Grain yield had a significantly positive correlation with yield per panicle, PW, straw weight, biomass yield, TKW, harvest index, and threshing percent at genotypic and phenotypic levels. The present investigation showed that day-to-flower, grain filling period, LL, PL, PW, biomass yield, TKW, and threshing percent exerted a positive direct effect on grain yield of sorghum both at the genotypic and phenotypic levels. Therefore, direct selection for these traits may prove effective for the improvement of grain yield in the germplasm under study. The residual effect (*h* = 0.2059) shows that traits that are included in the phenotypic path coefficient analysis explained 79.41% of the total variation in grain yield. The genotypic residual effect was low, indicating that the traits that were included in the genotypic path analysis explained 85% of the total variation in grain yield showing that the independent traits explained the dependent trait more than two-thirds. Therefore, the genotypic path coefficient analysis-based selection for sorghum improvement is very appropriate. This analysis confirmed that day-to-flower, grain filling period, and biomass yield produced a high positive direct effect on grain yield, which appeared to be the prominent traits when selecting grain yield in sorghum genotypes. Based on the findings of the study, it is suggested that day-to-flower, grain filling period, and biomass yield are key factors that directly impact grain yield in sorghum. These traits should be prioritized in breeding programs aimed at improving total yield in sorghum genotypes. Hence, moderate heritability coupled with moderate GA was observed for the grain filling period, yield per panicle, straw weight, biomass yield, grain yield, and threshing percent. Therefore, the findings of this study might be used as a source of information by researchers who are engaged in the areas of sorghum improvement programs. The results of the path coefficient analysis indicated that biomass yield, grain filling period, LW, and DF were the main determinants of grain yield in sorghum.

## Figures and Tables

**Table 1:** List of sorghum genotypes used for the study.

Genotype	Code	Genotype	Code	Genotype	Code	Genotype	Code	Genotype	Code	Genotype
26892	G1	69334-B	G22	70845-B	G43	74701	G64	204611	G85	
26899	G2	69335-A	G23	71010	G44	74684-B	G65	204615	G86	
26901	G3	69335-B	G24	74641	G45	74686	G66	204621	G87	
26905	G4	69337	G25	71076	G46	74688	G67	204622	G88	
27907	G5	69338	G26	69331	G47	74689-A	G68	204623	G89	
27909	G6	69339	G27	69332	G48	74689-B	G69	204624	G90	
27915	G7	69341	G28	69333	G49	74693	G70	204625	G91	
27916	G8	70046	G29	69334	G50	200613	G71	74642	G92	
27918	G9	70050	G30	69335	G51	200615-A	G72	74643	G93	
27915	G10	70052	G31	69336	G52	200615-B	G73	74645	G94	
27916	G11	70055	G32	69337	G53	200615-C	G74	74646	G95	
27918	G12	70056-A	G33	69338	G54	200617	G75	74647	G96	
27919	G13	70056-B	G34	69339	G55	201343	G76	74649	G97	
27922	G14	70063-A	G35	69340	G56	201348	G77	74651	G98	
27924	G15	70084	G36	69341	G57	201453	G78	74652	G99	
69319	G16	70161	G37	74666	G58	204601	G79	74653	G100	
69321	G17	70229	G38	74667	G59	204602	G80	74654	G101	
69323	G18	70568	G39	74669	G60	204604	G81	74655	G102	
69327	G19	70678	G40	74670	G61	204605-A	G82	74656	G103	
69332	G20	70229	G41	74675	G62	204605-B	G83	74657	G104	
69334-A	G21	70845-A	G42	74684-A	G63	204607	G84	74658	G105	
74660	G106	74688	G131	74685	G156	214010	G181	204611	G206	211192
74661	G107	74689	G132	74686	G157	214043	G182	204612	G207	211486
74663	G108	74690	G133	74687	G158	214044	G183	204613	G208	213004
74664	G109	74691	G134	74688	G159	214046	G184	204614	G209	213008
74665	G110	74693	G135	74689	G160	214056	G185	204615	G210	213017
74666	G111	74695	G136	74690	G161	214063	G186	204616	G211	Melkam
74667	G112	74698	G137	74691	G162	214079	G187	204617	G212	Dekeba
74669	G113	74699	G138	74693	G163	214109	G188	204618	G213	Meko
74670	G114	74700	G139	74695	G164	215025	G189	204619	G214	Macia
74671	G115	74701	G140	74698	G165	215026	G190	204620	G215	Gambella 1107
74672	G116	74702	G141	74699	G166	216906	G191	204621	G216	Red Swazi
74674	G117	74703	G142	74700	G167	216907	G192	204622	G217	Seredo
74675	G118	74704	G143	74701	G168	204610	G193	204623	G218	Gobiye
74676	G119	74705	G144	74702	G169	204608	G194	204633	G219	ESH-1
74677	G120	74706	G145	74703	G170	204609	G195	204634	G220	ESH-2
74678	G121	200613	G146	74704	G171	204610	G196	204635	G221	ESH-4
74679	G122	200614	G147	200617	G172	204611	G197	204636	G222	ESH-5
74680	G123	200615	G148	201343	G173	204612	G198	206094	G223	Teshale
74681	G124	200616	G149	201344	G174	204613	G199	206127	G224	Argity
74682	G125	200617	G150	201348	G175	204614	G200	206285	G225	Tilahu
74683	G126	201343	G151	201349	G176	204615	G201	206286		
74684	G127	201344	G152	201350	G177	204616	G202	210905		
74685	G128	201348	G153	213019	G178	204617	G203	210922		
74686	G129	201349	G154	213021	G179	204618	G204	211022		
74687	G130	201350	G155	213026	G180	204619	G205	211191		

*Note:* In the table, codes 1 to 2010 correspond to genotypes obtained from the Ethiopian Biodiversity Institute (EBI), while codes 2011 to 225 refer to released varieties obtained from the Melkassa Agricultural Research Center.

**Table 2:** Analysis of variance in simple lattice design and expected mean square.

Source of variation	Df	SS	MS	*F*-test	
				Obtained	Required 5% 1%
Total	rk^2^ − 1	SS_T_	MS_T_	—	
Replications	*r* − 1	SSr	MSr	MSr/*E*_*b*_	
Treatments (Unadj.)	*k* ^2^ − 1	SSt	MSt	MSt/Ee	
Block within replication (adj.)	*r*(*k* − 1)	SSb	*E*_*b*_ = SSb/*r*(*k* − 1)		
Intrablock error	(*k* − 1) (*rk*-*k*-1)	SSe	*E*_*e*_ = SSe/((*k* − 1) (*rk*-*k* − 1))		

*Note: r* = number of replicates of each treatment, *k* = number of units per block (block size), *t* = treatment, T = total, *b* = total number of blocks in the experiment, *E*_*b*_ = error for block, *E*_*e*_ = experimental error.

Abbreviations: Df = degree of freedom, MS = mean squares, and SS = sum squares.

**Table 3:** Analysis of variance (mean squares) for 17 quantitative traits studied on sorghum genotype at Jinka, in 2021.

Traits	Source of variation				LSD_0.05_	CV (%)
	Mean square of replication (Df = 1)	Mean square of genotype (Df = 224)	Blocks within replications (Df = 28)	Intrablock error (Df = 196)		
DF	2630.54⁣^∗∗^	81.35⁣^∗∗^	61.84	61.04	15.40	10.12
DM	179.24⁣^∗^	183.55⁣^∗∗^	151.94⁣^∗^	99.84	19.69	7.71
GFP	4183.08⁣^∗∗^	258.66⁣^∗∗^	259.29	162.36	25.11	23.34
PH	30.91^ns^	7239.76⁣^∗∗^	97.02^ns^	110.06	20.7	4.61
LN	0.01^ns^	4.87⁣^∗∗^	0.01^ns^	0.01	0.19	1.00
LL	1.50^ns^	234.27⁣^∗∗^	1.50^ns^	1.50	2.42	1.9
LW	1.14E − 13^ns^	2.79⁣^∗∗^	1.40E − 32^ns^	−5.80E − 16	0	0
LA	0.68^ns^	9489.99⁣^∗∗^	0.68^ns^	0.68	1.63	1.74
Pl	3.10^ns^	68.59^ns^	79.43^ns^	67.50	16.19	19.57
YP	292.96^ns^	945.64⁣^∗^	877.26^ns^	682.90	51.50	39.15
PW	2817.95^ns^	3220.71⁣^∗^	3767.46⁣^∗^	2370.52	95.95	25.18
SW	1706464⁣^∗^	906322⁣^∗∗^	595804	480012	1365.3	31.52
BM	12086979	8283953⁣^∗∗^	7250994	5549772	4642.36	29.19
TKW	18.20⁣^∗∗^	26.38⁣^∗∗^	10.23	15.32	7.71	13.01
GY	4710107	6710424⁣^∗∗^	5070203	4332684	4101.85	35.09
HI	0.001	0.013⁣^∗∗^	0.007	0.007	0.17	11.86
TP	21.60	183.97	112.32	128.66	22.35	32.76

*Note:* ns, ^∗^, ^∗∗^: significant, highly significant and nonsignificant at 0.05 and 0.01 probability level, based on *F*-test, respectively.

Abbreviations: BM = biomass yield (kg), DF = days to flowering, DM = days to maturity, GFP = grain filling period, GY = grain yield (kg), HI = harvest index (%), LA = leaf area (cm^2^), LL = leaf length, LN = leaf number, LSD = least significance difference, LW = leaf width, PH = plant height (cm), PL = panicle length (cm), PW = panicle weight (cm), SW = straw weight (kg), TP = threshing percent (%), and YP = yield per panicle (g).

**Table 4:** Estimates of mean, range, variance components, coefficients of variability, heritability, and genetic advance of the 17 quantitative traits of sorghum at Jinka, in 2021.

Traits	Mean ± SE	Range	*σ*_*g*_^2^	*σ*_*e*_^2^	*σ*_*ph*_^2^	PCV (%)	GCV (%)	*h* ^2^ *b* (%)	GA	GAM
DF	77.24 ± 0.43	63–100	10.16	61.04	71.2	10.92	4.13	14.27	2.48	3.22
DM	133.83 ± 0.64	110–152.5	36.94	99.84	136.78	8.74	4.54	27.01	6.52	4.87
GFP	56.58 ± 0.76	23.5–82	48.15	162.36	210.51	25.64	12.26	22.87	6.85	12.10
PH	225.79 ± 4.01	75–393	3564.85	110.1	3674.95	26.85	26.44	97.00	121.32	53.73
LN	9.45 ± 0.10	6–14	2.43	0.01	2.44	16.53	16.50	99.59	3.21	33.96
LL	64.53 ± 0.72	38–98	77.98	1.50	79.48	13.82	13.68	98.11	18.04	27.96
LW	0.95 ± 0.08	0.5–12	1390	−5.80E − 16	1390	3924.50	3924.50	100.00	76.91	8096.23
LA	47.25 ± 4.59	14.2–673.8	4744.65	0.68	4745.33	145.79	145.78	99.99	142.09	300.72
Pl	42.46 ± 0.39	23.5–58.98	0.55	67.50	68.05	19.43	1.75	0.81	0.14	0.32
Yp	68.03 ± 1.45	16.49–133.8	131.37	682.9	814.27	41.95	16.85	16.13	9.50	13.96
PW	201.43 ± 2.68	89.33–450.2	425.10	2370.52	2795.62	26.25	10.24	15.21	16.59	8.23
SW	2245.51 ± 44.88	768.1–5725	213155	480012	693167	37.08	20.56	30.75	528.17	23.52
BM	8250.26 ± 135.7	3338–14012	1367090.5	5549772	6916862.5	31.88	14.17	19.76	1072.36	13.00
TKW	29.30 ± 0.24	16–39	5.53	15.32	20.85	15.58	8.03	26.52	2.50	8.53
GY	6004.62 ± 122.1	1613–11511	1188870	4332684	5521554	39.13	18.16	21.53	1043.77	17.38
HI	0.72 ± 0.01	0.44–0.91	0.003	0.007	0.01	13.89	7.61	30.00	0.06	8.60
TP	34.35 ± 0.64	7.51–69.22	27.66	128.66	156.32	36.40	15.31	17.69	4.56	13.29

Abbreviations: BM = biomass yield (kg), DF = days to flowering, DM = days to maturity, GFP = grain filling period, GY = grain yield (kg), HI = harvest index (%), LA = leaf area (cm^2^), LL = leaf length, LN = leaf number, LW = leaf width, PH = plant height (cm), PL = panicle length (cm), PW = panicle weight (cm), SE = standard error, SW = straw weight (kg), TKW = thousand kernel weight (g), TP = threshing percent (%), and YP = yield per panicle (g).

**Table 5:** Phenotypic (above diagonal) and genotypic (below diagonal) association coefficients among 17 traits on sorghum.

	DF	DM	GfP	PH	LN	LL	LW	LA	Pl	Yp	PW	SW	BM	TKW	GY	HI	TP
DF		0.03	−0.54	0.03	0.02	−0.05	−0.07	−0.07	0.05	0.00	−0.03	0.01	0.01	−0.06	0.00	0.00	0.00
DM	−0.07		0.83	0.26	0.38	0.24	0.00	0.01	0.17	−0.04	−0.01	0.17	0.02	−0.24	−0.04	−0.21	−0.05
GFP	−0.67	0.79		0.20	0.31	0.23	0.04	0.05	0.12	−0.03	0.01	0.14	0.02	−0.18	−0.03	−0.18	−0.04
PH	−0.04	0.03	0.04		0.36	0.20	0.01	0.01	0.13	0.06	−0.01	0.24	0.14	−0.06	0.07	−0.14	0.08
LN	0.06	0.04	−0.01	0.00		0.07	0.03	0.02	0.03	0.04	0.10	0.26	0.12	−0.12	0.03	−0.23	−0.04
LL	0.06	0.04	−0.01	0.00	1.00		0.19	0.27	0.09	−0.05	−0.06	0.14	0.00	−0.09	−0.05	−0.17	0.01
LW	0.09⁣^∗^	0.03⁣^∗∗^	−0.28	0.17^ns^	−0.01	0.38⁣^∗^		0.99	0.02	−0.13	−0.14	−0.01	−0.12	0.11	−0.13	−0.14	−0.07
LA	0.06	0.04	−0.01	0.00	1.00	1.00	0.54⁣^∗^		0.02	0.13	0.07	0.06	0.14	−0.06	0.14	0.13	0.11
Pl	−0.03	−0.02	0.01	0.07	0.02	0.02	0.07^ns^	0.02		−0.23	−0.02	−0.03	−0.21	0.07	−0.22	−0.14	−0.20
Yp	0.07	−0.09	−0.12	0.07	0.13	0.13	0.02^ns^	0.13	−0.06		0.42	0.14	0.92	0.09	0.97⁣^∗^	0.60	0.77
PW	0.06	−0.06	−0.08	0.03	0.07	0.07	0.03^ns^	0.07	−0.07	0.48⁣^∗^		0.09	0.38	0.09	0.39⁣^∗^	0.21	−0.19
SW	−0.03	0.02	0.03	0.06	0.06	0.06	−0.03	0.06	−0.06	0.25	0.26		0.46	−0.09	0.14⁣^∗^	−0.63	0.11
BM	0.07	−0.08	−0.10	0.08	0.14	0.14	−0.42	0.14	−0.06	0.92⁣^∗^	0.49	0.55⁣^∗^		0.04	0.95⁣^∗^	0.35⁣^∗^	0.73⁣^∗^
TKW	0.07	−0.20	−0.19	0.03	−0.06	−0.06	0.22	−0.06	−0.06	0.12	0.08	0.14⁣^∗^	0.19		0.08	0.13	0.03
GY	0.09	−0.10	−0.13	0.08	0.14	0.14	−0.21⁣^∗^	0.14	−0.05	0.97⁣^∗^	0.48⁣^∗^	0.29⁣^∗^	0.96⁣^∗^	0.17⁣^∗^		0.62⁣^∗^	0.77⁣^∗^
HI	0.06	−0.14	−0.14	0.03	0.13	0.13	−0.24	0.13	0.02	0.61⁣^∗^	0.15⁣^∗^	−0.51⁣^∗^	0.37⁣^∗^	0.01	0.60⁣^∗^		0.51
TP	0.04	−0.06	−0.07	0.04	0.11	0.11	−0.10	0.11	−0.03	0.77⁣^∗^	−0.14	0.10	0.68⁣^∗^	0.06	0.75⁣^∗^	0.57	

*Note:* ^∗^, ^∗∗^, ns: significant, highly significant and nonsignificant at 0.05 and 0.01 probability level, respectively.

Abbreviations: BM = biomass yield (kg), DF = days to flowering, DM = days to maturity, GFP = grain filling period, GY = grain yield (kg), HI = harvest index (%), LA = leaf area (cm^2^), LL = leaf length, LN = leaf number, LW = leaf width, PH = plant height (cm), PL = panicle length (cm), PW = panicle weight (cm), SW = straw weight (kg), TKW = thousand kernel weight (g), TP = threshing percent (%), and YP = yield per panicle (g).

**Table 6:** Estimates of phenotypic regression analysis of the direct (bolded diagonal) and indirect (off-diagonal) effects of yield related traits on grain yield of sorghum genotypes evaluated at Jinka, 2021.

	DF	DM	GfP	PH	LN	LL	LW	LA	PL	Yp	PW	SW	BM	TKW	HI	TP	*r*_*ph*_
DF	**0.52**	−0.06	−0.67	−0.01	−0.01	−0.01	0.00	0.09	−0.04	0.00	0.00	0.00	−0.02	0.08	0.00	0.00	−0.13
DM	−0.06	**−1.00**	0.79	−0.01	0.00	0.00	0.00	−0.03	−0.03	0.00	0.00	0.00	0.03	−0.23	0.00	0.00	−0.54
GfP	−0.55	−0.79	**1.34**	0.00	0.03	−0.02	0.00	−0.04	0.01	0.00	0.00	0.00	0.04	−0.22	0.00	0.00	−0.21
PH	−0.01	−0.02	0.02	**0.40**	0.13	−0.01	0.00	0.00	0.01	−0.02	0.01	0.08	0.04	0.00	0.12	0.01	0.76
LN	0.00	−0.01	0.00	0.04	**0.03**	0.00	0.00	0.00	−0.01	−0.03	0.40	0.12	0.23	0.04	0.18	0.00	0.99
LL	−0.01	0.00	−0.01	−0.01	0.00	**0.02**	0.05	0.02	0.01	0.02	0.02	0.30	0.02	0.01	0.22	0.00	0.65
LW	−0.02	−0.03	−0.01	−0.01	0.00	0.00	**−0.30**	0.27	0.36	0.04	0.33	0.27	−0.33	0.00	0.25	0.00	0.82
LA	−0.02	−0.04	−0.01	0.01	0.02	0.03	−0.30	**0.22**	0.02	0.05	0.01	0.01	−0.25	0.06	0.21	0.00	0.03
PL	−0.55	−0.79	0.01	0.04	0.40	0.30	0.25	0.03	**0.12**	0.00	0.00	0.00	0.02	−0.07	0.00	0.00	−0.24
Yp	−0.03	0.02	−0.16	0.00	0.00	0.00	−0.16	0.20	0.00	**0.00**	0.00	0.00	−0.31	0.14	0.00	0.00	−0.29
PW	0.06	0.09	−0.11	0.00	0.24	0.30	0.36	0.24	0.00	0.00	**0.00**	0.00	−0.17	0.09	0.00	0.00	1.11
SW	0.05	0.06	−0.08	0.76	0.45	0.22	0.30	0.02	0.00	0.00	0.00	**−0.34**	−0.19	0.16	0.00	0.00	1.41
BM	−0.02	−0.02	−0.14	0.04	0.06	0.00	−0.11	0.22	0.00	0.00	0.00	−0.19	**1.15**	0.01	0.00	0.00	1.01
TKW	0.06	0.08	−0.26	−0.06	0.06	−0.02	−0.05	0.00	0.00	0.00	0.00	−0.05	0.22	**0.00**	0.00	0.00	−0.02
HI	0.06	0.20	−0.19	0.00	0.02	−0.04	−0.16	0.20	0.00	0.00	0.00	0.17	0.43	0.00	**0.00**	0.00	0.69
TP	0.05	0.14	−0.09	0.00	0.00	0.00	−0.08	0.09	0.00	0.00	0.00	−0.03	0.78	0.00	0.00	**0.00**	0.86

*Note:* Residual = 0.2059, *r*_*ph*_ = phenotypic correlation.

Abbreviations: BM = biomass yield (kg), DF = days to flowering, DM = days to maturity, GFP = grain filling period, GY = grain yield (kg), HI = harvest index (%), LA = leaf area (cm^2^), LL = leaf length, LN = leaf number, LW = leaf width, PH = plant height (cm), PL = panicle length (cm), PW = panicle weight (cm), SW = straw weight (kg), TKW = thousand kernel weight (g), TP = threshing percent (%), and YP = yield per panicle (g).

**Table 7:** Estimates of genotypic regression analysis of the direct (bolded diagonal) and indirect (off-diagonal) effects of yield related traits on grain yield of sorghum genotypes evaluated at Jinka, 2021.

	DF	DM	GfP	PH	LN	LL	LW	LA	Pl	Yp	PW	SW	BM	TKW	HI	TP	*r*_*g*_
DF	**0.70**	−0.03	−0.67	0.00	0.00	0.00	−0.07	0.07	0.00	0.00	0.00	−0.01	0.01	0.00	0.00	0.00	0.00
DM	0.02	**−1.06**	1.04	0.00	−0.01	0.02	0.00	−0.01	0.00	0.01	0.00	−0.09	0.03	0.00	0.02	0.00	−0.03
GfP	−0.38	−0.88	**1.25**	0.00	−0.01	0.02	0.04	−0.05	0.00	0.01	0.00	−0.07	0.03	0.00	0.02	0.00	−0.02
PH	0.02	−0.27	0.25	**−0.01**	−0.01	0.02	0.01	−0.01	0.00	−0.01	0.00	−0.13	0.19	0.00	0.01	0.01	0.07
LN	0.01	−0.41	0.39	0.00	**−0.02**	0.01	0.03	−0.02	0.00	−0.01	0.00	−0.14	0.17	0.00	0.02	0.00	0.03
LL	−0.03	−0.25	0.28	0.00	0.00	**0.09**	0.18	−0.27	0.00	0.01	0.00	−0.07	0.00	0.00	0.02	0.00	−0.04
LW	−0.05	0.00	0.05	0.00	0.00	0.02	**0.96**	−0.98	0.00	0.03	−0.01	0.01	−0.17	0.00	0.01	0.00	−0.13
LA	−0.05	−0.01	0.07	0.00	0.00	0.02	0.96	**−0.99**	0.00	0.03	−0.01	0.00	−0.15	0.00	0.01	0.00	−0.12
Pl	0.03	−0.19	0.15	0.00	0.00	0.01	0.02	−0.02	**0.00**	0.06	0.00	0.02	−0.29	0.00	0.01	−0.01	−0.21
Yp	0.00	0.04	−0.04	0.00	0.00	0.00	−0.12	0.12	0.00	**−0.24**	0.02	−0.07	1.27	0.00	−0.06	0.05	0.97
PW	−0.02	0.01	0.01	0.00	0.00	−0.01	−0.14	0.14	0.00	−0.10	**0.05**	−0.05	0.53	0.00	−0.02	−0.01	0.19
SW	0.01	−0.18	0.17	0.00	−0.01	0.01	−0.01	0.00	0.00	−0.03	0.00	**−0.53**	0.64	0.00	0.06	0.01	0.14
BM	0.00	−0.02	0.02	0.00	0.00	0.00	−0.11	0.11	0.00	−0.22	0.02	−0.24	**1.38**	0.00	−0.03	0.01	0.82
TKW	−0.04	0.26	−0.22	0.00	0.00	−0.01	0.10	−0.10	0.00	−0.02	0.00	0.05	0.06	**0.00**	−0.01	0.00	0.07
HI	0.00	0.22	−0.22	0.00	0.01	−0.02	−0.14	0.14	0.00	−0.14	0.01	0.33	0.49	0.00	**−0.09**	0.03	0.62
TP	0.00	0.06	−0.05	0.00	0.00	0.00	−0.06	0.06	0.00	−0.19	−0.01	−0.06	1.01	0.00	−0.05	**0.07**	0.78

*Note:* Residual = 0.15, *r*_*g*_ = genotypic correlation.

Abbreviations: BM = biomass yield (kg), DF = days to flowering, DM = days to maturity, GFP = grain filling period, GY = grain yield (kg), HI = harvest index (%), LA = leaf area (cm^2^), LL = leaf length, LN = leaf number, LW = leaf width, PH = plant height (cm), PL = panicle length (cm), PW = panicle weight (cm), SW = straw weight (kg), TKW = thousand kernel weight (g), TP = threshing percent (%), and YP = yield per panicle (g).

## Data Availability

The data used to support the findings of this study are made available from the corresponding author upon reasonable request.
